# Preoperative fMRI predicts memory decline following anterior temporal lobe resection

**DOI:** 10.1136/jnnp.2007.115139

**Published:** 2007-09-26

**Authors:** H W R Powell, M P Richardson, M R Symms, P A Boulby, P J Thompson, J S Duncan, M J Koepp

**Affiliations:** 1 Department of Clinical and Experimental Epilepsy, Institute of Neurology, University College London, London, UK and MRI Unit, National Society for Epilepsy, Chalfont St Peter, UK; 2 Department of Clinical Neuroscience, Institute of Psychiatry, Kings College London, London, UK

## Abstract

**Background:**

Anterior temporal lobe resection (ATLR) benefits many patients with refractory temporal lobe epilepsy (TLE) but may be complicated by material specific memory impairments, typically of verbal memory following left ATLR, and non-verbal memory following right ATLR. Preoperative memory functional MRI (fMRI) may help in the prediction of these deficits.

**Objective:**

To assess the value of preoperative fMRI in the prediction of material specific memory deficits following both left- and right-sided ATLR.

**Methods:**

We report 15 patients with unilateral TLE undergoing ATLR; eight underwent dominant hemisphere ATLR and seven non-dominant ATLR. Patients performed an fMRI memory paradigm which examined the encoding of words, pictures and faces.

**Results:**

Individual patients with relatively greater ipsilateral compared with contralateral medial temporal lobe activation had greater memory decline following ATLR. This was the case for both verbal memory decline following dominant ATLR and for non-verbal memory decline following non-dominant ATLR. For verbal memory decline, activation within the dominant hippocampus was predictive of postoperative memory change whereas activation in the non-dominant hippocampus was not.

**Conclusion:**

These findings suggest that preoperative memory fMRI may be a useful non-invasive predictor of postoperative memory change following ATLR and provide support for the functional adequacy theory of hippocampal function. They also suggest that fMRI may provide additional information, over that provided by neuropsychology, for use in the prediction of postoperative memory decline.

Anterior temporal lobe resection (ATLR) may lead to seizure freedom in at least 60% of patients with medically refractory temporal lobe epilepsy (TLE) and is now being carried out earlier and in those with less severe epilepsy.^1^ The hippocampus and medial temporal structures play an important role in memory encoding^2^ and ATLR carries a risk of memory impairment. With improved patient selection, cases of dense amnesia are fortunately rare,^3^ nevertheless more subtle, but clinically important, memory deficits remain common. Patients undergoing unilateral ATLR are at risk of a decline in verbal memory following surgery involving the language dominant hemisphere^4^ and a decline in topographical memory following non-dominant temporal lobe resection.^5^

Prognostic indicators for material specific memory decline following ATLR include the severity of hippocampal sclerosis (HS) on MRI, with less severe left HS increasing the risk of verbal memory decline,^6^ and preoperative memory performance, with better performance increasing the risk of memory decline.^7^^–^10 Memory decline has also been found to correlate inversely with the severity of HS in the resected hippocampus, with patients with more severe HS having less memory decline.^11^ The intracarotid amytal test (IAT) has been advocated for the prediction of postoperative memory deficits but has a number of disadvantages, notably the fact that it is an expensive invasive procedure.^12^

Functional MRI (fMRI) has potential for replacing the IAT and for providing additional data to those provided by baseline neuropsychological assessment. Recent studies have suggested that fMRI may help to predict memory decline following ATLR. Previous work in our group showed that greater verbal memory encoding activity in the left hippocampus compared with the right hippocampus predicted the extent of verbal memory decline following left ATLR.^13^ Rabin *et al* used a complex visual scene encoding task that causes symmetrical medial temporal lobe (MTL) activation in controls to demonstrate a correlation between MTL activation asymmetry ratios and post-surgical memory outcome, with increased activation ipsilateral to the seizure focus correlating with greater memory decline.^14^ Janszky *et al* used Roland’s Hometown Walking test in patients with right TLE, and demonstrated a correlation between preoperative fMRI asymmetry index during this task and postoperative change in non-verbal memory following right ATLR.^15^

We previously demonstrated a material specific lateralisation of function in the MTL in controls^16^ and evidence of some reorganisation of function to the contralateral MTL in patients with TLE.^17^ Contralateral reorganisation of function was not as effective as ipsilateral hippocampal function as correlations between fMRI activation and baseline memory performance showed that patients using their ipsilateral, to-be-resected hippocampus had better memory performance than those with encoding reorganised to the opposite side. In this study, we report results of preoperative fMRI and preoperative and postoperative neuropsychological assessment in individual patients with left and right TLE undergoing ATLR. We hypothesised that greater ipsilateral fMRI activation would be associated with greater verbal memory decline following ATLR resection on the language dominant side and greater non-verbal memory decline following non-dominant ATLR.

## PATIENTS AND METHODS

### Subjects

We studied 15 consecutive patients (median age 36 years (range 22–47); seven females) with medically refractory TLE undergoing ATLR at the National Hospital for Neurology and Neurosurgery, London, UK. All patients had undergone structural MRI at 1.5 T.^18^ Of the seven patients with left TLE, six had HS and one had a MTL dysembryoblastic neuroepithelial tumour (DNET). Of the eight patients with right TLE, six had HS, one had a MTL DNET and one a MTL glioma. Video-EEG had confirmed seizures arising from the ipsilateral medial temporal lobe in all 15. Hippocampal volumetry was carried out demonstrating a normal contralateral hippocampus in all subjects.^18^ All patients were receiving antiepileptic medication which remained unchanged at the time of postoperative neuropsychological testing and all were fluent English speakers. Handedness was determined using a standardised questionnaire. Language dominance was assessed using a range of fMRI tasks,^19^ revealing left hemisphere dominance in all but one patient. This patient (patient No 10) was left-handed and confirmed to be right hemisphere dominant for language on both fMRI and IAT. His pattern of neuropsychological test results, with a decline in verbal memory score and little change in non-verbal memory, were also in keeping with resection of the language dominant hemisphere. All patients underwent standardised neuropsychological assessment before and after operation.^20^

Patient demographics, neurological test results and surgical outcome data are detailed in table 1. The ILAE classification of postoperative seizure outcome following epilepsy surgery was used.^21^ Preoperative and postoperative neuropsychological test results are detailed in table 2. The study was approved by the National Hospital for Neurology and Neurosurgery and the Institute of Neurology Joint Research Ethics Committee and informed written consent was obtained from all subjects.

**Table 1:** Clinical and demographic data for the 15 patients

Patient No	Age/sex	Handedness	Epilepsy onset (y)	Seizure types and frequency (per month)	Post-op outcome (ILAE class.)	Duration of follow-up (months)	MRI and pathological diagnosis	Clinical and EEG	RHV (cm^3^)	LHV (cm^3^)	TIV (cl)	HVR (%)	AEDs (mg/day)	Language dominance
1	25/F	Right	17	CPS 8 SGTC 0.5	1	42	Left HS	Left TLE	2.729	1.351	168.5	50	TPR 150 LTG 200	Left
2	37/M	Left	1	SPS 12 CPS 4	2	40	Left HS	Left TLE	3.3	1.88	175	57	VPA 800 CBZ 800 LVT 2000	Left
3	33/M	Right	1	SPS 4 CPS 4	1	36	Left HS	Left TLE	2.83	2.02	NM	71	PMD 500 CBZ 1200 CLB 10 TPR 175	Left
4	37/F	Right	18	CPS 10	1	24	Left HS	Left TLE	2.912	1.602	143.5	55	CBZ 1200 GBP 2700	Left
5	28/M	Right	3	CPS 1	1	36	Left HS	Left TLE	3.077	1.417	171.2	46	LVT 3000	Left
													LTG 600	
6	31/M	Right	10	CPS 50 SGTC 3	1	NA	Left MTL DNET	Left TLE	3.731	3.797	187.2	98	CBZ 1200 CLN 1.5 LTG 100	Left
7	37/F	Right	1	SPS 12 CPS 8 SGTC 1	3	30	Left HS, left fusiform gyrus ganglioglioma	Left TLE	2.8	2.385	134.8	85	CBZ 1000 CLB 10	Left
8	47/M	Right	13	CPS 1	2	36	Right HS	Right TLE	1.038	2.769	145.9	37	LVT 500 PHT 300 CBZ 800	Left
9	44/F	Right	14	CPS 4	1	30	Right HS	Right TLE	2.606	3.034	154.9	86	LVT 750 PHT 400 CLB 10	Left
10	36/M	Left	15	SPS 6 CPS 6 SGTC 3	1	24	Right MTL glioma	Right TLE	3.118	2.774	152.3	89	CBZ 1600 CLB 20 LTG 400	Right
11	46/F	Right	8	CPS 5	1	24	Right HS	Right TLE	1.756	2.695	147	65	TPR 600 PMD 1000 OXC 2400	Left
12	32/F	Right	19	CPS 2	4	36	Right HS	Right TLE	2.299	2.562	139.5	90	CBZ 1600 LVT 1000	Left
13	29/M	Right	9	CPS 30 SGTC 4	3	36	Right HS	Right TLE	2.273	2.978	162.9	76	VPA 2400	Left
14	22/M	Left	18	CPS 3 SGTC 0.33	1	32	Right MTL DNET	Right TLE	2.493	2.554	168.4	98	VPA 2000 GBP 600	Left
15	41/F	Right	14	CPS 4	1	NA	Right HS	Right TLE	1.628	2.302	150.8	71	TGB 15	Left

AED, antiepileptic drug; CBZ, carbamazepine; CLB, clobazam; CLN, clonazepam; CPS, complex partial seizure; DNET, dysembryoplastic neuroepithelial tumour; EEG, electroencephalogram; GBP, gabapentin; HS, hippocampal sclerosis; HVR, hippocampal volume ratio of affected to unaffected side; LHV, left hippocampal volume; LTG, lamotrigine; LVT, levetiracetam; MTL, medial temporal lobe; NA, not available; NM, not measured; OXC, oxcarbazepine; PHT, phenytoin; PMD, primidone; RHV, right hippocampal volume; SGTC, secondary generalised tonic–clonic seizure; SPS, simple partial seizure; TGB, tiagabine; TIV, total intracranial volume; TLE, temporal lobe epilepsy; TPR, topiramate; VPA, sodium valproate.

**Table 2:** Neuropsychological data for the 15 patients

Patient No	Resection side	VIQ	PIQ	Preop verbal learning	Postop verbal learning	Verbal learning change	Preop design learning	Postop design learning	Design learning change
1	Left	82	80	73	53	−20*	87	56	−31*
2	Left	94	93	57	39	−18*	51	42	−9
3	Left	86	88	60	47	−13	80	95	+15
4	Left	105	110	72	64	−8	76	82	+6
5	Left	76	81	52	40	−12	58	62	+4
6	Left	91	99	48	48	0	55	82	+27
7	Left	70	94	80	63	−17*	53	75	+22
8	Right	73	86	51	56	+5	27	29	+2
9	Right	95	104	79	65	−14	80	56	−24
10	Right	82	99	63	41	−22*	57	64	+7
11	Right	88	84	66	57	−9	36	67	+31*
12	Right	87	110	56	51	−5	80	41	−39*
13	Right	93	114	68	73	+5	64	73	+9
14	Right	93	92	52	59	+7	76	66	−10
15	Right	90	88	69	45	−24*	31	42	+11

PIQ, performance intelligence quotient; VIQ, verbal intelligence quotient.

*Subjects with a significant memory change, as identified using reliable change indices.

### Neuropsychological tests used

The measures of memory employed in this study are standard in our surgical programme. In the verbal learning task, the subject is read a list of 15 words five times and on each presentation has to attempt to recall as many of the words as possible. The percentage of correct responses was used as a measure of verbal memory efficiency. For non-verbal memory, we used the design learning task; the subject is presented with a design on five occasions with recall being tested after each presentation. The percentage of correct responses over the five trials was used as a second measure of non-verbal memory efficiency.

Each memory test was repeated 3 months after surgery in each patient, and measures of memory change following surgery were calculated as preoperative verbal learning–postoperative verbal learning, and preoperative design learning–postoperative design learning. These measures of memory change were then used to test for correlations between preoperative fMRI activation and memory change following ATLR. Patients with a clinically significant postoperative decline were identified using reliable change indices (RCIs).^22^ The RCI (80% confidence interval) was 16 for verbal learning and 28 for design learning.

We also tested for correlations between preoperative hippocampal volume and memory change following surgery. For this, and all subsequent group analyses, the patients were divided according to whether they underwent dominant or non-dominant hemisphere ATLR. The patient with right TLE and right language dominance was therefore included in the dominant group along with all patients with left TLE. We calculated Pearson’s correlation coefficient between left (dominant) hippocampal volume and change in verbal memory following dominant ATLR, and between right (non-dominant) hippocampal volume and change in non-verbal memory following non-dominant ATLR. Finally, we tested for correlations between preoperative memory performance and memory change following surgery. We calculated the Pearson’s correlation coefficient between preoperative verbal learning and change in verbal learning following dominant ATLR, and between preoperative design learning and change in design learning following non-dominant ATLR.

### MR data acquisition

MRI studies were performed on a 1.5 T General Electric Signa Horizon scanner. Standard imaging gradients with a maximum strength of 22 mT/m and slew rate of 120 T/m/s were used. All data were acquired using a standard quadrature birdcage head coil for both RF transmission and reception. For each subject we acquired a whole brain high resolution EPI image with whole brain coverage. These were spatially normalised and mean images of patients undergoing dominant and non-dominant resections were calculated upon which to display results.

For the memory task, gradient echo, echo planar T2* weighted images were acquired, providing blood oxygenation level dependent contrast. Each volume comprised 12 contiguous 2.3 mm oblique axial slices through the temporal lobes, with a 22 cm field of view, 96×96 matrix and inplane resolution of 1.72×1.72 mm. TE was 40 ms and TR 4.5 s. The field of view was positioned to cover the temporal lobes with the anteroposterior axis aligned with the long axis of the hippocampus on sagittal views with the body of the hippocampus in the centre. The imaging time series was realigned, normalised into standard anatomical space and smoothed with a Gaussian kernel of 10 mm full width half maximum. Standard SPM normalisation (using an affine transformation and non-linear registration) was performed using the high resolution whole brain EPI.

### Psychological task

Stimuli of three different material types (pictures (P), words (W) and faces (F)) were visually presented to the subjects during a single scanning session. The pictures were black and white nameable line drawn objects,^23^ the words were single concrete nouns and the faces were black and white photographs unfamiliar to the subjects. A total of 210 stimuli were presented, one every 4 s, in seven cycles. Each cycle consisted of a block of 10 pictures, a block of 10 words and a block of 10 faces (each lasting 40 s) followed by 20 s of crosshair fixation. Subjects performed a deep encoding task which involved making a judgement on whether each stimulus was pleasant or unpleasant,^24^ but were not explicitly instructed to subsequently remember the items. This task was used in order to encourage stimulus encoding, but the type of response was not used in any subsequent parts of the fMRI analysis. Sixty minutes after scanning, subjects performed a recognition test which was not scanned; this comprised three blocks, one for each of the three material types. During each recognition block, the 70 stimuli of each type presented during scanning were randomly mixed with 35 foils and presented in a manner identical to that used during scanning. Subjects were instructed to indicate whether they could remember seeing each stimulus during scanning (R response) or whether it was new (N response).

The 210 encoding stimuli that had been presented during scanning were then classified according to the responses made during the recognition test. A correct (R) response indicated the stimulus was subsequently remembered while an incorrect (N) response indicated the stimulus was subsequently forgotten. For each of the three stimulus types (P, W and F), R and N responses were identified, giving a total of six event types: PR, PN, WR, WN, FR and FN. These were then entered as regressors in the design matrix. To calculate recognition accuracy, stimuli seen in the recognition test were classified as “hits” (stimuli correctly remembered) and “false alarms” (foils incorrectly tagged as remembered). Recognition accuracy was calculated for each stimulus type as: hit rate–false alarm rate.

### Data analysis

Imaging data were analysed using Statistical Parametric Mapping (SPM2).^25^ To test for subsequent memory effects, an event related design was used to compare encoding related responses to individual stimuli that were subsequently remembered versus stimuli that were forgotten.^26^ A two level, random effects analysis was used. At the first level, trial specific responses were modelled for each subject by convolving a delta function that indicated each event onset with the canonical haemodynamic response function to create regressors of interest, one regressor for each of the six event types described above. Each subject’s movement parameters were included as confounds. Parameter estimates pertaining to the height of the haemodynamic response function for each regressor of interest were calculated for each voxel. Contrasts of parameter estimates were calculated in a voxel-wise manner to produce, for each subject, one contrast image corresponding to the subsequent memory effect for each material type (PR minus PN, WR minus WN, FR minus FN) and an overall memory encoding effect for each subject (R minus N, collapsed across all three stimulus types). All of these images were used for the second level analysis.

### “Difference image” analysis

For each subject, we created “difference images” by rotating the normalised contrast images by 180° in the x axis and subtracting this “flipped” image from the original contrast image.^13^ This created images of encoding asymmetry for each stimulus type showing left minus right activation in the left hemisphere and right minus left activation on the right.

At the second level of the random effects analysis, simple regression was used within each group to look for brain regions showing correlations between preoperative encoding asymmetry and both verbal and non-verbal memory change following surgery. We report all MTL activations that survived an uncorrected threshold of p<0.001, and were of a minimum cluster size of 30 voxels. The uncorrected threshold was adopted because of the low signal-to-noise ratio in the anterior temporal lobe^16^ ^27^ and as we were testing a specific hypothesis regarding MTL activation. MTL regions of activation were labelled with reference to Duvernoy’s *The Human Hippocampus*.^28^ In addition, we carried out a small volume correction and all reported regions survived the small volume correction at p<0.05 at both the voxel and cluster level.

### Region of interest analysis

We defined spherical (10 mm radius) regions of interest (ROIs) for the left and right hippocampus. The left-sided ROI was centred on the anterior hippocampal region previously reported to show a correlation between encoding asymmetry and change in verbal memory following left ATLR.^29^ A homotopic ROI was created on the right. We used the fMRI parameter estimates within the left and right ROIs, and calculated Pearson’s correlation coefficient between hippocampal fMRI and change in memory following dominant and non-dominant ATLR.

## RESULTS

### Memory change following surgery

#### Resection in language dominant hemisphere (n = 8)

Memory test data are summarised in table 2 with patients showing a significant memory decline highlighted. Seven of the eight patients undergoing dominant ATLR had a postoperative decline in verbal learning score, four of which were significant. The remaining patient had no change. The mean change between preoperative and postoperative verbal learning score was −14, ranging from 0 to −22 (p = 0.001). One patient also had a significant decline in design learning score. The mean change between preoperative and postoperative design learning score was 5, ranging from an increase of 27 to a decline of 31 (p = 0.46).

#### Resection in non-dominant hemisphere (n = 7)

Only one patient undergoing non-dominant ATLR had a significant postoperative decline in design learning score, and in one there was a significant increase. The mean change between preoperative and postoperative design learning score was −3, ranging from an increase of 31 to a decline of 39 (p = 0.76). One patient also had a significant decline in verbal learning score. The mean change between preoperative and postoperative verbal learning score was −5, ranging from an increase of 7 to a decline of 24 (p = 0.3).

### Correlations between hippocampal volume and postoperative memory change

There were no statistically significant correlations between hippocampal volume and verbal memory decline in the dominant ATLR patients or between hippocampal volume and non-verbal memory decline in the non-dominant ATLR patients.

### Correlations between preoperative memory performance and postoperative memory change

There were no statistically significant correlations between preoperative verbal learning and verbal memory decline in the dominant ATLR patients. In the non-dominant ATLR patients a significant correlation was seen between preoperative design learning and non-verbal memory decline (Pearson’s correlation coefficient −0.751; p = 0.03), characterised by a greater decline in patients with better preoperative memory function.

### Correlations between preoperative fMRI and postoperative memory change

#### Difference images

**Figure 1:**
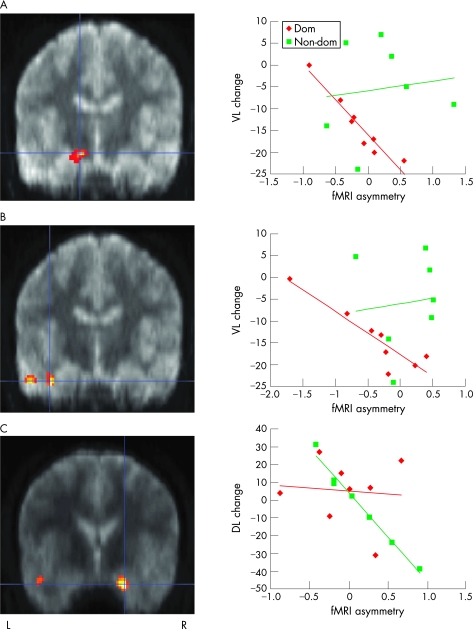
Relationships between preoperative functional MRI (fMRI) activation (arbitrary units) and postoperative memory change for words (A), overall memory (B) and faces (C) in patients undergoing anterior temporal lobe resection (ATLR). Regions showing a significant correlation between fMRI activation asymmetry and postoperative change in verbal learning (VL) are superimposed onto the normalised mean EPI image from patients undergoing dominant (A, B) and non-dominant (C) resections. Left (L) and right (R) side of the brain are indicated. The correlation at the peak voxel is illustrated graphically on the right for both the dominant ATLR group (red line) and the non-dominant ATLR group (green line). For patients undergoing dominant ATLR, significant correlations were seen between hippocampal fMRI activation asymmetry for words (A; MNI coordinates −14 −14 −16, Z score 3.9, cluster size 35 voxels) and overall memory (B; MNI coordinates −38 −14 −28, Z score 3.69, cluster size 39 voxels), and postoperative change in verbal learning. For patients undergoing non-dominant ATLR, a significant correlation was seen between amygdala fMRI activation asymmetry for faces (C; MNI coordinates 22 −2 −26, Z score 3.95, cluster size 52 voxels) and postoperative change in design learning (DL).

In patients undergoing dominant ATLR, correlations were seen between left–right difference in hippocampal encoding activation for words (r^2^ = 0.92, p*<*0.001, fig 1A) as well as for overall memory encoding (r^3^ = 0.93, p*<*0.001, fig 1B), and decline in verbal learning score. No correlations were seen between fMRI activation in these regions and change in verbal learning score in patients undergoing non-dominant ATLR (fig 1A, B).

In patients undergoing non-dominant ATLR a correlation was observed between right–left difference in amygdala encoding activation for faces and change in design learning score (r^2^ = 0.98, p<0.001, fig 1C). In patients undergoing dominant ATLR, no correlations were seen between fMRI activation in this region and change in memory (fig 1C).

#### ROI analysis

**Figure 2:**
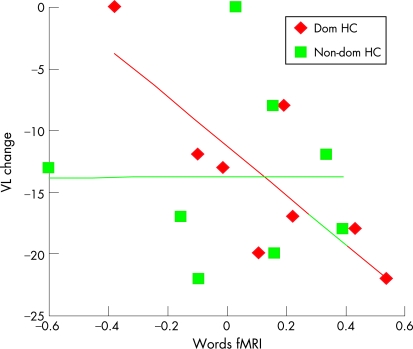
Relationship between preoperative functional MRI (fMRI) activation and postoperative memory change within dominant and non-dominant hippocampal (HC) regions of interest. For patients undergoing dominant anterior temporal lobe resection, a significant correlation was seen between dominant hippocampal encoding related fMRI activation for words and postoperative decline in verbal learning (VL) (red line). No significant correlation was seen in the non-dominant hippocampus (green line).

In patients undergoing dominant ATLR there was a significant correlation between dominant hippocampal encoding activation for words and postoperative change in verbal learning score (Pearson’s correlation coefficient = −0.811, p = 0.015, fig 2), characterised by a greater decline in verbal memory in patients with greater activation within the dominant hippocampus. No correlation was seen in the non-dominant hippocampus (Pearson’s correlation coefficient = −0.004, p = 0.992, fig 2).

In the patients undergoing non-dominant ATLR, no significant correlation was seen between right hippocampal encoding activation for faces or pictures and postoperative change in design learning.

## DISCUSSION

In patients with unilateral TLE performing an fMRI test of memory encoding, relatively greater ipsilateral compared with contralateral MTL activation predicted declines in both verbal and non-verbal memory following dominant and non-dominant hemisphere ATLR. Furthermore, for dominant hemisphere resections, activation within the ipsilateral hippocampus was predictive of postoperative memory change with greater hippocampal activation for word encoding being correlated with greater verbal memory decline following surgery. No such correlation was observed in the contralateral hippocampus.

This study has a number of strengths. Firstly, the event related analysis allowed us to specifically test for subsequent memory effects rather than make an assumption of memory encoding. Secondly, we used an fMRI paradigm that tested both verbal and non-verbal memory in a single scanning session, allowing us to specifically test clinically relevant, material specific memory function. Thirdly, we found correlations between fMRI activation during this paradigm and changes in memory performance, as measured on standard epilepsy surgery neuropsychological tests and accordingly findings relate to clinically identified memory decline. Finally, in addition to showing correlations between asymmetry of MTL activation, we found that activation in the ipsilateral, to-be-resected hippocampus was of most relevance in predicting postoperative material specific memory deficits.

We demonstrated previously a material specific lateralisation of memory encoding in the medial temporal lobes in healthy controls; with word encoding seen in the left MTL, picture encoding relatively bilateral and face encoding being mainly on the right.^16^ As a number of other memory fMRI studies had only observed activation in posterior hippocampal and parahippocampal structures, separate from the areas normally resected during ATLR,^30^^–^34 we optimised our fMRI acquisition and paradigm to visualise activation in the anterior hippocampal and MTL, regions that would be resected during ATLR.^16^ We then demonstrated in patients with unilateral HS that increased activation in the damaged hippocampus correlated with better memory performance, while increased activation in the contralateral hippocampus correlated with worse performance.^17^ This suggested that while there was evidence for functional reorganisation to the opposite hippocampus, this was a less efficient process, incapable of preserving good memory function.

Our findings relate to a relatively small sample of patients and will require confirmation in larger groups. In addition, the sample size does not allow us to investigate the influence of other potentially important factors such as underlying pathology, and the duration and severity of epilepsy on our findings. However, our results replicate and extend previous fMRI findings in an independent patient sample, and are consistent with the clinical picture of patients undergoing ATLR, increasing our confidence in the validity of the findings.

Our findings corroborate and extend findings from previous studies. In a group of patients with left HS, relatively greater activation for word encoding in the left hippocampus compared with the right hippocampus predicted the extent of verbal memory decline following left ATLR.^13^ Regression analysis demonstrated that greater activation within a left hippocampal ROI predicted a greater postoperative decline in verbal memory, a similar finding to this study. Right hippocampal activation however also predicted postoperative verbal memory outcome,^29^ a finding not supported in our current study.

In a study of patients with right TLE, a correlation was found between an asymmetry index of MTL fMRI activation and decline in non-verbal memory following right ATLR, characterised by increased right- compared with left-sided activity being associated with greater memory decline.^15^ In a group of patients with left and right TLE, asymmetry ratios of MTL activation during a visual scene encoding task were correlated with memory outcome.^14^ Increased ipsilateral activation was inversely correlated with memory outcome and no correlation was seen in the contralateral hippocampus. The measure of memory outcome used was the change between pre- and post-surgical scene recognition performance, likely to be more sensitive to right MTL pathology, rather than a more standard neuropsychological measure of memory.

Two different models of hippocampal function have been proposed to explain memory deficits following ATLR; hippocampal reserve and functional adequacy.^35^ According to the functional adequacy model, it is the capacity of the hippocampus that is to be resected that determines whether changes in memory function will be observed, while the hippocampal reserve theory suggests that postoperative memory decline depends on the capacity or reserve of the contralateral hippocampus to support memory following surgery. Activation asymmetry measures do not distinguish between whether it is retained ipsilateral or lack of contralateral activation that is the principal risk factor for memory decline following ATLR (ie, whether the functional adequacy or hippocampal reserve model holds true). Our finding that greater activation in the damaged hippocampus correlates with greater postoperative memory decline, with no significant correlation demonstrated contralaterally, along with our previous finding that increased activation in the damaged hippocampus correlated with better baseline memory performance^17^ supports the functional adequacy model of hippocampal function. We only tested memory 3 months following surgery which may be too early for any hippocampal reserve to become fully functional. It is possible that retesting patients a year following surgery would reveal some recovery in memory function and provide more support for the hippocampal reserve model.

Clinically, it is what happens to individual patients that is important and it is noteworthy that one of our non-dominant ATLR cases showed a larger postoperative decline in verbal memory than the dominant ATLR cases, and a second patient had a verbal memory decline of 14 points. From fig 2A it can be seen that both of these patients had greater hippocampal activation on the right than on the left (left minus right activation is negative) for the verbal memory encoding task. It is not easy to explain why these two patients with right TLE should be primarily using the right hippocampus for verbal memory. Declines in verbal memory have been reported by other investigators following right ATLR and this serves to highlight that the two hippocampal systems may not work as independently as has been suggested by the material specific hypothesis.^36^ For example, visual strategies such as imagery may be used to enhance verbal memory competence and it may be that the verbal memories of individuals making use of such strategies preoperatively may be disproportionately affected by right ATLR. Alternatively, the presence of additional pathology in the left MTL, not seen on neuroimaging, may lead to patients using their right hippocampus for verbal memory function preoperatively. Of the cases showing significant decline in verbal memory following right ATLR in this study, all were rendered seizure free by surgery and there were no electroclinical features to suggest left MTL pathology. Whatever the reason, our finding suggests that fMRI is able to provide important predictive information, above and beyond that provided by baseline neuropsychology; namely that removing the functional area, regardless of its site, leads to a decline in function.

When assessing the clinical utility of any new predictor of memory change it is important to consider whether a change in score is significant, or meaningful, to the individual patient. RCIs have been reported as one way of identifying patients with a clinically significant postoperative decline.^22^ The RCI (80% confidence interval) is 16 for verbal learning and 28 for design learning, therefore all patients with a postoperative score of 16 (verbal learning) or 28 (design learning) lower than their preoperative score may be classified as having a significant decline. The numbers of patients in this study do not permit conclusions as to whether patients with a significant memory decline differed from others, in the day-to-day impact of the changes. Assessment of this issue is complicated further by studies showing that subjective memory complaints bear little relation to formal neuropsychological test scores and may be more closely related to levels of anxiety and depression.^37^ It does not appear that newer definitions of memory change such as RCIs are any more closely allied with the subjective experience of postoperative change than traditional measures.

Relatively greater right compared with left amygdala activation for face encoding predicted non-verbal memory decline following non-dominant ATLR but no correlation was observed with right amygdala activation alone. The amygdala has been implicated in the processing of fearful expressions.^38^ Impaired recognition of fearful facial expressions has been shown in patients with bilateral amygdala damage.^39^ Some of the faces included in this study had fearful expressions and we previously demonstrated that face encoding in normal controls was associated with right amygdala activation.^16^ Given that the amygdala has been shown to be involved in face encoding and is resected during ATLR, it is not surprising that activation within this structure correlates with postoperative memory change.

The ability to predict postoperative verbal and non-verbal memory deficits for individual patients following left and right ATLR is an important part of the presurgical evaluation. Current predictors of postoperative memory outcome include the severity of hippocampal sclerosis on preoperative structural imaging with larger hippocampal volume being correlated with a decline in verbal memory following resection^6^ and preoperative memory performance on neuropsychological testing, with better performance increasing the risk of clinically significant memory decline.^7^^–^10 Given the small number of subjects in this study, it is not a surprise that we did not see any correlations between hippocampal volume and memory outcome. Nevertheless, fMRI did predict memory changes in this sample and indicates that fMRI activation is not merely an indirect marker of hippocampal atrophy. While there was no correlation between preoperative verbal memory and postoperative decline, we did observe a correlation between preoperative non-verbal memory performance and postoperative decline.

The postoperative memory changes observed in this study are in keeping with the view that decline in verbal memory following dominant ATLR is clinically more important than decline in non-verbal memory following non-dominant ATLR. It is therefore encouraging that it was in these patients that fMRI was of the greatest predictive value. We anticipate that the use of fMRI paradigms of memory encoding, as used in our study, in combination with baseline neuropsychology and structural MRI, will enable preoperative prediction of material specific memory impairments seen following unilateral ATLR to be made with greater accuracy.
